# Technological and Organoleptic Parameters of Craft Beer Fortified with Powder of the Culinary–Medicinal Mushroom *Pleurotus eryngii*

**DOI:** 10.3390/jof9101000

**Published:** 2023-10-09

**Authors:** Fortunato Cirlincione, Antonino Pirrone, Ignazio Maria Gugino, Aldo Todaro, Vincenzo Naselli, Nicola Francesca, Antonio Alfonzo, Giulia Mirabile, Valeria Ferraro, Gaetano Balenzano, Maria Letizia Gargano

**Affiliations:** 1 Department of Agricultural, Food and Forest Sciences, University of Palermo, Viale delle Scienze, Bldg. 5, 90128 Palermo, Italy; fortunato.cirlincione@unipa.it (F.C.); antonino.pirrone@unipa.it (A.P.); ignaziomaria.gugino@unipa.it (I.M.G.); aldo.todaro@unipa.it (A.T.); vincenzo.naselli@unipa.it (V.N.); nicola.francesca@unipa.it (N.F.); antonio.alfonzo@unipa.it (A.A.); 2 Department of Pharmacy—Pharmaceutical Sciences, University of Bari “Aldo Moro”, University Campus “Ernesto Quagliariello”, Via E. Orabona 4, 70125 Bari, Italy; 3 Department of Soil, Plant, and Food Sciences, University of Bari “Aldo Moro”, Via Amendola 165/A, 70126 Bari, Italy; marialetizia.gargano@uniba.it

**Keywords:** beer, craft beer, *Pleurotus eryngii*, medicinal mushrooms, novel beverages, organoleptic profile, flavor, aroma, taste

## Abstract

Beer is one of the oldest and most popular alcoholic beverages and is currently consumed worldwide. The various components used in the brewing process have a physiological impact on the consumer and current research aims to improve its technological and functional properties through the addition of natural compounds (plants or mushrooms). In this work, the addition of two different amounts (5 and 10 g/L) of *Pleurotus eryngii* var. *eryngii* in powder form added at different production stages (PRE and POST alcoholic fermentation) showed the improvement in yeast viability during the alcoholic fermentation, increased the alcoholic content, and improved the sensorial profile. Regarding the organoleptic profile in the experimental samples, cocoa/chocolate and mushroom aromas were found and the samples PRE10 and POST5 received the best ratings with respect to all evaluated parameters.

## 1. Introduction

Beer is one of the world’s oldest and most popular alcoholic beverages and is currently consumed all over the world [1]. The Sumerians reported the first production of a beer-like drink around 6000 years ago [2]. The beer making process starts with the wort preparation which is made from raw materials which provide sugar as glucose and maltose to yeasts which perform the alcoholic fermentation (AF) [3]. Barley malt is mainly used for preparation but according to the recipe, other cereals can be added as wheat (raw or malted), corn, oat, and rice [4]. Beer can be considered a nutritionally valuable product because the various components used in the manufacturing process have physiological effects on consumption. It is characterized by a high carbohydrate content as well as the presence of protein, amino acids, vitamins, organic acids, microelements, and antioxidants [5]. However, despite being the most widely consumed alcoholic beverage in the world, beer is the subject of constant research focused on improving its technological aspects [6,7], raw material [8], and on the development of non-conventional beers [9].

Craft breweries have traditionally added fruits and spices to the brewing process to enhance the flavor and aroma of various beer styles [10]. Functional beers are unconventional beers that try to combine moderate alcoholic beverage consumption with health benefits [11]. This aim is in line with the emerging trend in the functional food market which attracts customers based on well-established trends in today’s society of disease prevention through a functional diet [12].

**Figure 1:**
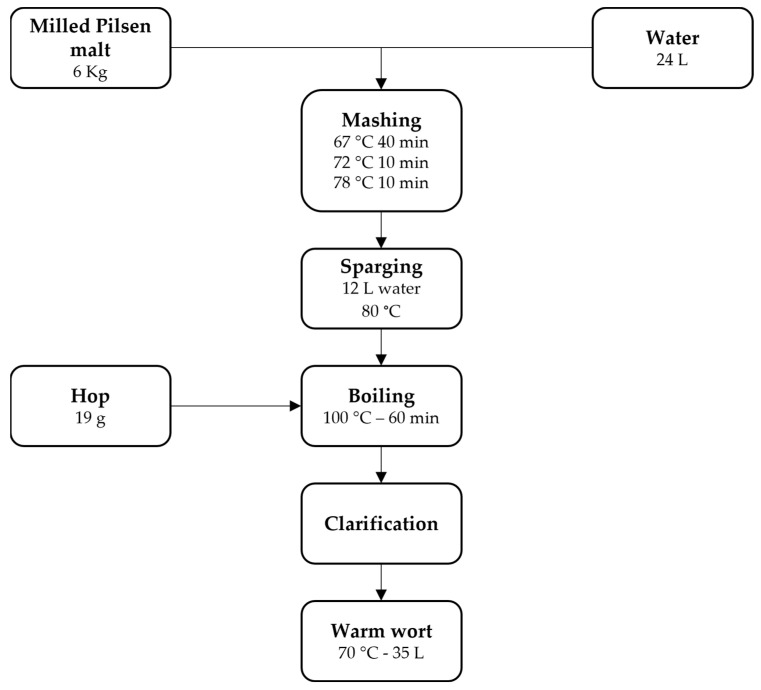
Wort production flow chart.

**Figure 2:**
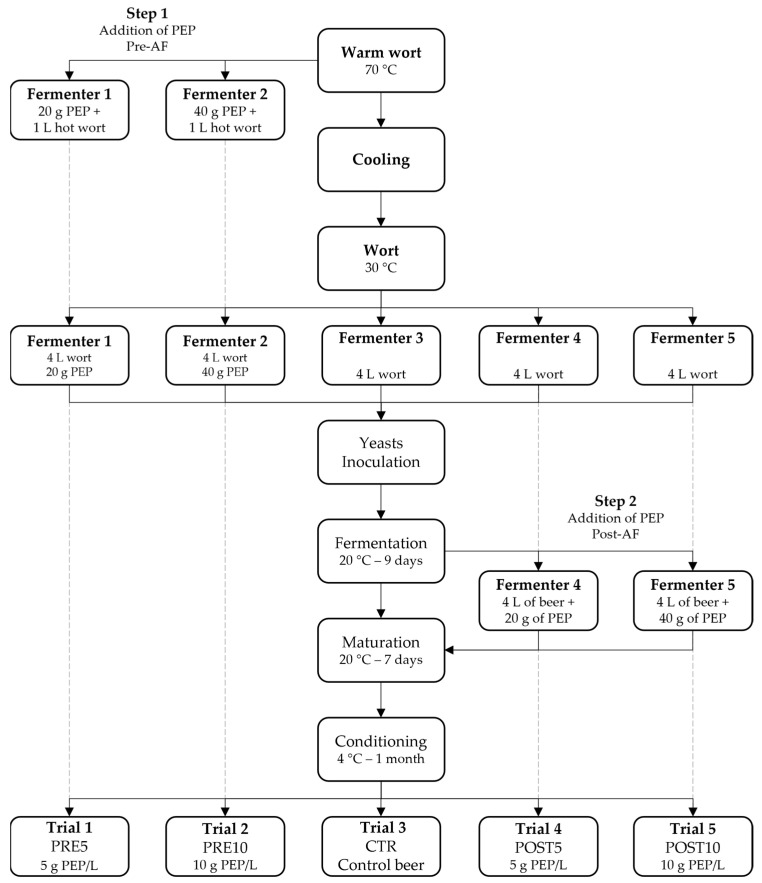
Experimental trial production flow chart.

In recent years, the food industry’s efforts have focused on adapting to consumer trends toward products with functional characteristics that can improve and prevent diet-related diseases [13] by adding naturally derived components or extracts to conventional products [14,15,16]. Recently, there has been much interest in the addition of food- and medicinal-interest mushroom powders and extracts into foods that are commonly consumed [16,17,18,19]. Mushrooms are known not only for their high nutritional value but also because they possess peculiar flavor, aroma, and aromatic compounds [20]. Various molecules contribute to the aroma composition of mushrooms, the most important among them being terpenoids that are used in food and cosmetics industries [21]. In particular, over the past decade, we have focused our attention toward the *Pleurotus eryngii* species complex which comprises high-quality edible mushrooms that grow on the roots of various Apiaceae plants [22]. Recent investigations based on the evaluation of morphological and ecological characters combined with molecular analysis have provided a new explanation for this critical taxonomic group. The largest cluster (*P. eryngii* s. str.) was subdivided into taxa at the variety level: *P. eryngii* (DC.) Quél var. *eryngii*, *P. eryngii* var. *ferulae* Lanzi, *P. eryngii* var. *thapsiae* Venturella, Zervakis and Saitta and *P. eryngii* var. *elaeoselini* Venturella, and Zervakis and La Rocca [23]. In this paper, we analyzed the influence that the addition of powdered *P. eryngii* var. *eryngii* (PEP), an edible and medicinal mushroom, brings on the physical, chemical, and sensory characteristics of craft beer. Two different amounts of powder were added (5 g/L and 10 g/L) in two different stages of production (before and at the end of AF) as reported in flow charts (Figure 1 and Figure 2). The main hypothesis of this research was to develop an innovative product with distinctive sensory characteristics. Based on our knowledge, this is the first work reporting the use of the mushroom *P. eryngii* var. *eryngii* in the brewing process of craft beer.

## 2. Materials and Methods

### 2.1. Mushroom Material

*P. eryngii* var. *eryngii* basidiomes were collected in October 2022 in Basilicata (southern Italy) on grasslands via root residues of *Eryngium campestre* L. The collected samples were transported to the laboratory under refrigerated conditions (4 °C) and were subsequently identified and described morphologically. The herbarium samples are kept in the Herbarium of the Department of Agricultural and Forest Sciences of the University of Palermo (SAF). Under a laminar flow hood, a piece of flesh of basidioma was put, aseptically, in a Petri dish which contains potato dextrose agar (PDA) medium and incubated for 7 days at 26 ± 1 °C. After subsequent purification steps, the strain was stored in the Mycotheca of the Department (SAF). The strain number is C-143 and similar in productivity to the previously tested C-142 of similar geographical origin [24].

### 2.2. Substrate Preparation and Mushroom Cultivation

A piece of purified mycelium of *P. eryngii* var *eryngii* was used for spawn preparation by inoculation of wheat seeds that had been previously soaked in distilled water, placed in 1 L jars, and sterilized at 121 °C for 20 min. The substrate was made from wheat straw and wheat bran, moistened, mixed, and then transferred in heat-resistant polypropylene bags model XLS-T (Unicorn bags, Plano, TX, USA) equipped with a filter (cut-off 0.2 µm). Each bag, weighing 4 kg, was sterilized at 121 °C for 1 h and, after cooling, was inoculated with spawn under aseptic conditions. The inoculated bags were sealed with manual impulse bag sealer (Tecnopack corporation, Sunrise, FL, USA) and placed in an heratherm IGS60 incubator (Thermo Fisher Scientific, Waltham, MA, USA) at 26 ± 1 °C in dark conditions. After 60–80 days, the mycelium had completely colonized the substrate and was considered ready for basidiomata production that was carried out in a department greenhouse. After collection, the basidiomes were sliced and dried by using a stainless steel Valla air drier (Borgotaro, Parma, Italy), pulverized with Vorwerk Bimby^®^ blender (Vorwerk and Co. KG, Wuppertal, Germany), and stored at 4 °C in vacuum-sealed bags until required.

### 2.3. Brewing Raw Materials and Beer Production

The traditional brewing process involves the use of water, barley malt, hops, and yeast. Brewing was performed in the pilot plant of the Agricultural and Forest Sciences of the University of Palermo (Italy) by using an “all-in-one” microbrewery plant Klarstein model 10,031,629 (Chal-Tec GmbH, Berlin, Germany), as reported in Figure 1. In total, 6 kilograms of Pilsen malt (BestMalz, Heidelberg, Germany) was ground using a two-roller mill (Brouwland, Beverlo, Belgium) and soaked in 24 L of water in which the pH had previously been corrected by the addition of 6 g of CaSO_4_ and 6 g of CaCl_2_ as reported by Marconi et al. [25]. The mash was heated to 67 °C for 40 min to perform single-phase mash until complete sugar conversion, which was verified with an iodine solution test. Subsequently, the mixture was heated up for 10 min at 72 °C and 10 min at 78 °C. The cereal grains were washed (sparged) with 12 L H_2_ O (80 °C) for a total volume of 36 L. The wort was then boiled for 60 min during which time 19 g of Hallertau Magnum hop pellets (Mr. Malt^®^, Pasian di Prato, Italy) were added to obtain a final concentration of 25 IBU (international bitter units). The final volume after boiling was 35 L with 10 °Bx (Brix degree). The wort was then clarified in a whirlpool for 10 min of recirculation and 10 min of rest [25] until it reached 70 °C. The produced wort showed the following characteristics: 5.18 pH, 10 °Bx, 1048 SG (Specific Gravity), 8.756 g/L of D-glucose, 0.657 g/L of D-fructose, 19.231 g/L of sucrose, and 33.167 g/L of maltose.

An aliquot of 70 °C of warmed wort was put into 5 L fermenters (1 L for each) containing 20 g and 40 g of PEP, respectively, to break down the bacterial load present in the raw material and avoid undesired fermentation. After wort cooling, the fermenters were filled to 4 L to reach the PEP concentration of 5 g/L (PRE5) and 10 g/L (PRE10), respectively. A total of 5 fermenters filled with 4 L of wort were prepared and inoculated as reported in flow chart (Figure 2).

Each fermenter was inoculated with a commercial strain of *Saccharomyces cerevisiae* SafAle™ US-05 (Fermentis, Lesaffre, France) at approximately 2 × 10^6^ CFU/mL [26] and incubated at 20 °C. At the end of the AF (day 9), the last 2 trials by addition of 20 g and 40 g of PEP corresponding to concentration of 5 g/L (POST5 sample) and 10 g/L (POST10 sample) were prepared. All trials were matured an additional 7 days after the end of AF (day 16) before being conditioned and bottled as described by Matraxia et al. [9]. All trials were produced in duplicate at two different times.

### 2.4. Sampling and Monitoring of Alcoholic Fermentation

The samples were taken at several points during the brewing process including wort, after the yeast strains inoculation (day 0), during the AF (day 2–day 9), during maturation (day 12–day 16), and after bottle conditioning. Each sample analysis was carried out in triplicate no later than 24 h after collection. The monitoring of yeast loads was carried out on Wallerstein Laboratory (WL) nutrient agar medium (Oxoid, Basingstoke, UK) and incubated at 25 °C for 48 h in aerobic conditions. All samples were serially diluted (1:10 ratio) in Ringer’s solution before being spread onto plates.

### 2.5. Physico-Chemical Parameters of Worts and Beers

All samples were subjected to pH measurement which was analyzed by a pH70 vio FOOD (XS Instruments, Carpi, Italy) pH meter while a DBR salt (Zetalab srl, Padova, Italy) refractometer was used to determine the Brix degree value. The determination of sugars (D-glucose, D-fructose, sucrose, and maltose), acetic acid, and glycerol of wort were performed by enzymatic determination as described by Matraxia et al. [9]; all chemicals and standards were bought from R-Biopharm AG (Darmstadt, Germany) and to respect the calibration curve of analyzer iCubio iMagic M9 (Shenzhen iCubio Biomedical Technology Co. Ltd. Shenzhen, China), appropriate sample dilution was carried out.

Real extract, wort extract, apparent extract, alcohol, real degree of attenuation, energy, specific gravity, density, and pH of beers after conditioning were determined by BeerFoss™ FT Go (FOSS A/S, Hillerød, Denmark).

The beer’s color was determined by spectrophotometry according to method 8.5 of the Analytica European Brewery Convention [27]. The beer samples were degassed in an ultrasonic bath at room temperature for 5 min and filtered through a syringe filter (0.45 µm, PVDF). The samples were diluted until the absorbance value was less than 0.8. The absorbance of beer sample was measured at wavelengths of 430 nm in a 10 mm cuvette. The value in EBC units was obtained by multiplying the absorbance by an appropriate factor.

### 2.6. Sensory Evaluation

The sensory evaluation of experimental beers was performed in a tasting room and in blind tasting conditions by twelve judges (from 27 to 46 years old) with backgrounds on sensory analysis, recruited at SAAF Department of the University of Palermo, and included a quantitative descriptive analysis to determine the color, odor, taste, and overall quality.

The sensory analysis of beers was conducted following the methodology reported by Matraxia et al. [9]. The panelists have evaluated 35 descriptive attributes related to aspect (color intensity and opacity), odor (intensity, complexity, fruity, floral, mushroom, cocoa, hoppy, malty/grainy, honey/caramel, acetic, oxidized/aged, sulphury, alcohol, DMS, and brine), taste (intensity, complexity, sweet, bitter, sour, astringent, fruity, mushroom, cocoa, spicy, hoppy, salty malty/grainy, roasted/burnt, body, oxidized/aged, DMS, and brine), and overall characteristics (visual, odor, taste, and overall satisfaction). Panelists also developed a consensus descriptive form for the evaluation of experimental beers, in which adjectives were paired with an unstructured 10-cm scale with the phrases “none/weak” and “strong” anchored at the left and right extremities, respectively [28].

The samples of beers (about 50 mL each) were provided at 16 °C in tasting glasses that respect the standard ISO type labelled with random codes. Between beers, water was available for rinsing. Each examination was made in a separate booth between 10:00 and 12:00 a.m. [29]. The results were calculated using the appropriate statistical analysis as the mean of three evaluations.

### 2.7. Statistical Analyses

Microbiological, physicochemical, and sensorial data were tested for differences using the one-way analysis of variance (ANOVA; general linear model) followed by the *post hoc* Tukey’s multiple range test applied for pairwise comparison. Statistical significance differences between samples was attributed for *p* ≤ 0.05 using XLStat^®^ add-in ver. 2014.5.03 (Addinsoft) for Microsoft Excel^®^.

## 3. Results and Discussion

### 3.1. Monitoring of Alcoholic Fermentation

**Figure 3:**
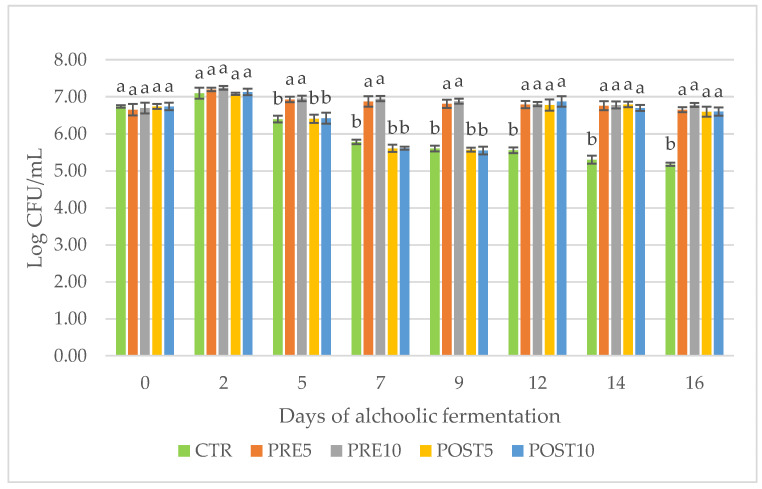
Monitoring of yeast loads during fermentation. CTR, wort without mushroom powder; PRE5, wort with 5 g/L of mushroom powder added before fermentation; PRE10, wort with 10 g/L of mushroom powder added before fermentation; POST5, wort with 5 g/L of mushroom powder added after fermentation; POST10, wort with 10 g/L of mushroom powder added after fermentation. Different superscript letters indicate that significant differences on yest load were displayed at each sampling time according to Tukey’s test for *p* < 0.05.

The yeast loads evaluated during the different steps of the AF were reported in Figure 3. Before inoculums into wort, the yeasts were under the detection limit. Following the yeast addition, the loads ranged between 6.65 and 6.74 Log CFU/mL. After 2 days of fermentation, the yeast load increased by 0.55 and 0.54 log cycles in PRE5 and PRE10, respectively, (where PEP was already added) and between 0.34–0.39 log cycles in other trials but no statistical differences were found. In general, after 2 days of AF, yeast loads reached similar levels (7.08–7.24 Log CFU/mL) to those reported by Pirrone et al. [10]. Between day 5 and day 9 of AF, the differences between the samples with and without PEP were most striking and statistically significant with higher values of yeast loads in PRE5 and PRE10 trials. In particular, yeast loads were constant in PRE5 and PRE10 samples (between 6.95 and 6.81 Log CFU/mL) while they decreased (from approx. 6.42 to approx. 5.55 Log CFU/mL) in POST5, POST10, and CTR samples in which PEP was not present. After day 9 sampling (end of AF), PEP was added in the POST5 and POST10 samples, as reported in MM. The yeast loads in samples POST5 and POST10 reached values (6.78 and 6.88 Log CFU/mL, respectively) similar to those found in samples PRE5 and PRE10 (6.79 and 6.81 Log CFU/mL, respectively) on day 12. This enhanced growth may be attributed to carbohydrates present in the fruiting body of *P. eryngii* [21,30] that may be available to yeast. A similar trend was reported in papers in which the addition of fruit juice causes increased yeast growth during fermentation [10,31,32]. The number of yeast cells was stable at the following sampling times (day 14 and day 16) in both PRE and POST samples and gradually decreased from 5.56 Log CFU/mL (day 12) to a value of 5.18 Log CFU/mL (day 16) in CTR sample.

### 3.2. Evolution of Chemical Parameters during Wort Alcoholic Fermentation

The chemical analysis of wort during AF showed differences between trials in D-fructose, D-sucrose, D-glucose, D-maltose, acetic acid, and glycerol contents (Table 1). At the beginning of AF (day 0), the samples that contained PEP (PRE5 and PRE10) showed a higher significant content of D-glucose, D-maltose, acetic acid, and glycerol than the samples without PEP. Regarding D-glucose, PRE5 and PRE10 were, respectively, 8.935 and 8.926 g/L significantly higher than that detected in samples without PEP, which ranged between 8.756 and 8.765 g/L. A progressive decrease in D-glucose content was recorded in the CTR, PRE5, and PRE10 samples from day 2 to the end of monitoring while in the POST5 and POST10 samples this occurred until day 9 when PEP was added. In the day 12 sample, the glucose content recorded was 0.270 g/L in POST5 and 0.492 g/L in POST10 samples. At the end of monitoring (day 16), the residual glucose content was 0.034 g/L in the CTR, 0.045 in PRE5, and 0.057 g/L in PRE10 while higher values were found in the POST5 and POST10 samples (0.224 g/L and 0.473 g/L, respectively). Similar behavior was observed in relation to the sucrose content which, at the end of monitoring, showed a content of 0.386 g/L in the POST5 sample and 0.830 g/L in the POST10 sample. Mushrooms are known to be a rich source of nutrients [33]; mushrooms belonging to the genus *Pleurotus* are particularly characterized by a high content of sugars, which can be more than 65% of dry weight [34,35].

Glycerol was absent in the first two sampling days in the samples where PEP is not present (CTR and POST samples) while it was detected at a concentration of 0.04 g/L in both PRE samples at day 0 and at day 2 and this reached 0.870 and 0.886 g/L (PRE5 and PRE10, respectively). From day 5 to the end of sampling, no significant differences were found between all samples and the detected quantity ranged between 0.850–0.873 g/L. In beers, glycerol is generally between 1–3 g/L [4] and in fermented alcoholic beverages it is the main component of the body attribute [36] but it also enhances flavor intensity and has an influence on aroma volatility [37,38]. Acetic acid is one of the organic acids that yeast can produce during the brewing process and can affect the organoleptic characteristics of the products [39]. Although significant differences were found in the acetic acid content of different trials, the value is significantly lower than that found by other authors [10,40]. The low percentage of acetic acid is interesting from a sensory point of view as acetic acid is commonly blamed for an unpleasant taste in beers, especially a sour vinegary flavor [40].

### 3.3. Physicochemical Properties of Beers

The main physicochemical properties of beers after bottling are reported in Table 2. All beers with the addition of PEP showed significantly higher alcohol content than the control beer. This is due to the sugars contained in PEP that could be used by the yeast during fermentation [41,42]. Similarly, the pH and color value in the beers containing PEP was significantly higher than in the CTR beer with the highest values in the samples containing 10 g/L PEP (PRE10 and POST10). All the experimental samples showed higher wort extract values than control beer and, as reported by Carvalho et al. [31], consequently higher alcohol content. Other authors have noted similar characteristics following the addition of vegetal material in beers. Gasiński et al. [43] found an increased alcohol content following the addition of mangoes in beer while Xu et al. [44] observed an increment of alcohol content, pH, and color value following the addition of fresh or dried okra.

### 3.4. Sensorial Evaluation

**Figure 4:**
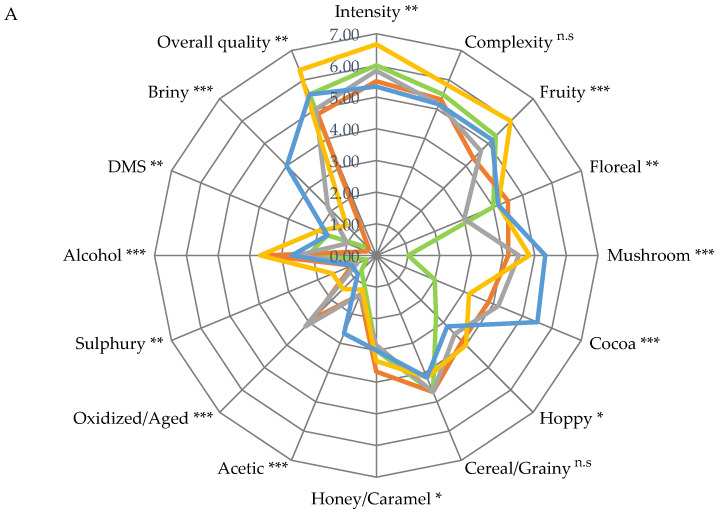

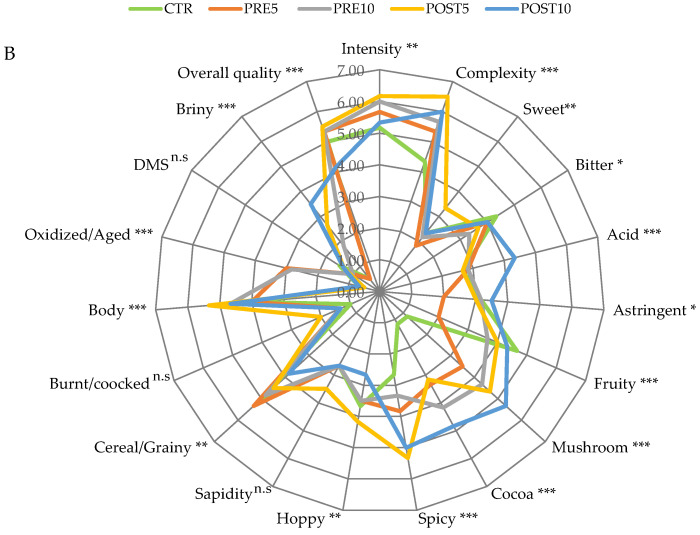
Spider plot of the sensory analysis performed on beers. (**A**) Odor; (**B**) Taste. Beer samples: CTR, beer without mushroom powder; PRE5, beer with 5 g/L of mushroom powder added before fermentation; PRE10, beer with 10 g/L of mushroom powder added before fermentation; POST5, beer with 5 g/L of mushroom powder added after fermentation; POST10, beer with 10 g/L of mushroom powder added after fermentation. *p* value: *, *p* < 0.05; **, *p* < 0,01; ***, *p* < 0.001; n.s., not significant.

**Figure 5:**
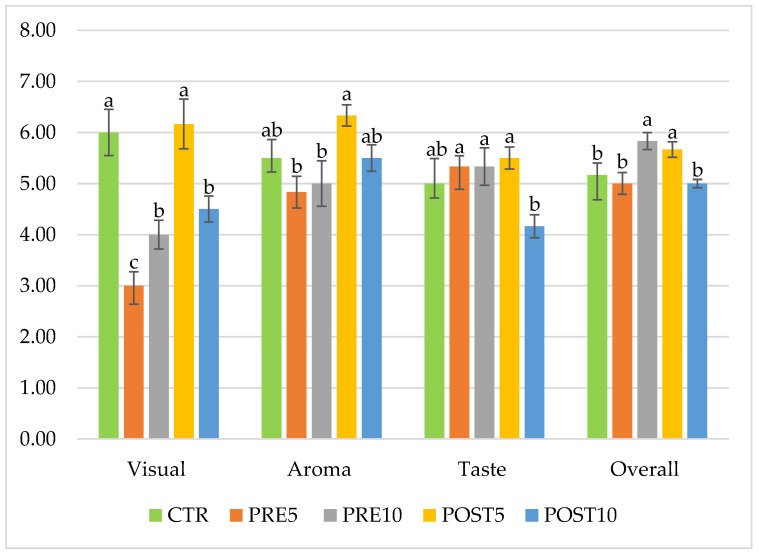
Visual, aroma, taste, and overall scores of beers. CTR, beer without mushroom powder; PRE5, beer with 5 g/L of mushroom powder added before fermentation; PRE10, beer with 10 g/L of mushroom powder added before fermentation; POST5, beer with 5 g/L of mushroom powder added after fermentation; POST10, beer with 10 g/L of mushroom powder added after fermentation. Different superscript letters indicate significant differences in scores performed at each attribute according to Tukey’s test for *p* < 0.05.

The sensorial evaluation of the experimental beers showed several significant differences between trials, as reported in Figure 4A,B. No differences were found in terms of complexity and cereal/grainy in aroma attributes and in sapidity, burt/cooked, and DMS in taste attributes. All samples that contained PEP showed cocoa and mushroom taste and aroma. In the PRE5 and PRE10 samples, the judges found an oxidized/aged taste and flavor while in POST10 briny an acetic off flavor and briny and acid off taste were found. In general, the POST5 sample received the highest score in terms of visual, taste, aroma, and overall acceptance. PRE10 scored the same as POST5 in overall acceptance and taste but scored lower in aroma and visual perception. Similar results were reported by Leskosek-Cukalovic et al. [45] in beers fortified with *Ganoderma lucidum* extract where the experimental beers showed better body, taste, and overall impression than the control beers. As reported in previous paragraphs, mushrooms belonging to the *Pleurotus* genus are rich in sugars which were utilized by the yeasts, increasing the alcohol content in the experimental beers containing PEP, which, together with glycerol and residual sugars, is primarily responsible for the “body” attribute of the beer [46]. The overall ratings of the different attributes evaluated in the trials are shown in Figure 5.

## 4. Conclusions

In this work, we investigated the effects of powdered *P. eryngii* var. *eryngii* on the physical, chemical, and sensory properties of craft beer. The PEP addition was carried out in two phases of manufacturing (before and after AF) and two different quantities of powder (5 g/L and 10 g/L). PEP is rich in nutrients [21,30], particularly carbohydrates, that can be used by yeast for their metabolism and this probably resulted in a statistically significant increase in yeast loads in all trials in which it was added. This hypothesis is confirmed by the higher amount of D-glucose and maltose detected in the trials in which PEP was added at the beginning of AF (PRE5 and PRE10). PEP-containing samples, both PRE and POST, showed a higher alcohol content, EBC color unit, and pH value than the control. The addition of PEP had a positive impact on the taste–olfactory characteristics of the beer evidenced by sensory analysis. The highest aroma production compared to the control occurred in the POST5 trial, which was the most highly rated in the sensory analysis in all parameters evaluated. In conclusion, the initial hypothesis of this research, i.e., to develop an innovative product with unique sensory characteristics was proven. Further analysis could be carried out by testing different wort conditions and by trying to assess the possible application to different beer styles.

## Figures and Tables

**Table 1:** Evolution of chemical parameters of wort during alcoholic fermentation.

	CTR	PRE5	PRE10	POST5	POST10	S.S.
**D-fructose (g/L)**						
Day 0	0.657 ± 0.006 ^a^	0.637 ± 0.038 ^a^	0.644 ± 0.027 ^a^	0.658 ± 0.006 ^a^	0.656 ± 0.006 ^a^	N.S.
Day 2	0.135 ± 0.004 ^a^	0.087 ± 0.001 ^b^	0.079 ± 0.003 ^b^	0.137 ± 0.004 ^a^	0.136 ± 0.004 ^a^	***
Day 5	0.018 ± 0.005 ^a^	0.023 ± 0.002 ^a^	0.022 ± 0.002 ^a^	0.018 ± 0.004 ^a^	0.018 ± 0.003 ^a^	N.S.
Day 7	0.032 ± 0.003 ^a^	0.022 ± 0.001 ^b^	0.023 ± 0.003 ^b^	0.03 ± 0.003 ^ab^	0.033 ± 0.003 ^a^	*
Day 9	0.018 ± 0.003 ^a^	0.007 ± 0.001 ^b^	0.003 ± 0.001 ^b^	0.018 ± 0.003 ^a^	0.018 ± 0.003 ^a^	***
Day 12	0.008 ± 0.001 ^c^	0.022 ± 0.002 ^a^	0.018 ± 0.004 ^ab^	0.022 ± 0.003 ^a^	0.011 ± 0.002 ^bc^	**
Day 14	0.019 ± 0.004 ^a^	0.015 ± 0.005 ^ab^	0.011 ± 0.003 ^abc^	0.009 ± 0.001 ^bc^	0.005 ± 0.001 ^c^	**
Day 16	0.022 ± 0.004 ^a^	0.013 ± 0.004 ^b^	0.010 ± 0.005 ^bc^	0.006 ± 0.001 ^bc^	0.002 ± 0.001 ^c^	**
**D-sucrose (g/L)**						
Day 0	19.231 ± 0.149 ^a^	19.066 ± 0.140 ^a^	19.368 ± 0.198 ^a^	19.380 ± 0.149 ^a^	19.235 ± 0.149 ^a^	N.S.
Day 2	0.250 ± 0.003 ^b^	0.354 ± 0.027 ^a^	0.225 ± 0.017 ^b^	0.255 ± 0.003 ^b^	0.258 ± 0.003 ^b^	***
Day 5	0.067 ± 0.004 ^c^	0.088 ± 0.005 ^b^	0.128 ± 0.004 ^a^	0.070 ± 0.004 ^c^	0.066 ± 0.004 ^c^	***
Day 7	0.058 ± 0.003 ^b^	0.101 ± 0.002 ^ab^	0.131 ± 0.037 ^a^	0.060 ± 0.003 ^b^	0.062 ± 0.003 ^b^	***
Day 9	0.080 ± 0.001 ^b^	0.116 ± 0.005 ^a^	0.122 ± 0.028 ^a^	0.080 ± 0.004 ^b^	0.080 ± 0.004 ^b^	**
Day 12	0.058 ± 0.003 ^c^	0.086 ± 0.004 ^c^	0.101 ± 0.005 ^c^	0.386 ± 0.003 ^b^	0.879 ± 0.042 ^a^	***
Day 14	0.052 ± 0.003 ^c^	0.070 ± 0.005 ^c^	0.101 ± 0.004 ^c^	0.479 ± 0.039 ^b^	0.884 ± 0.034 ^b^	***
Day 16	0.054 ± 0.004 ^c^	0.075 ± 0.002 ^c^	0.092 ± 0.004 ^c^	0.458 ± 0.047 ^b^	0.830 ± 0.020 ^a^	***
**D-glucose (g/L)**						
Day 0	8.756 ± 0.028 ^b^	8.935 ± 0.030 ^a^	8.926 ± 0.027 ^a^	8.760 ± 0.028 ^b^	8.765 ± 0.027 ^b^	***
Day 2	0.106 ± 0.002 ^a^	0.120 ± 0.002 ^a^	0.122 ± 0.016 ^a^	0.106 ± 0.005 ^a^	0.106 ± 0.004 ^a^	N.S.
Day 5	0.043 ± 0.005 ^c^	0.054 ± 0.003 ^b^	0.076 ± 0.002 ^a^	0.044 ± 0.005 ^bc^	0.045 ± 0.005 ^bc^	***
Day 7	0.034 ± 0.002 ^c^	0.060 ± 0.003 ^b^	0.077 ± 0.001 ^a^	0.037 ± 0.002 ^c^	0.036 ± 0.002 ^c^	***
Day 9	0.069 ± 0.002 ^b^	0.083 ± 0.001 ^a^	0.089 ± 0.004 ^a^	0.071 ± 0.002 ^b^	0.067 ± 0.002 ^b^	***
Day 12	0.037 ± 0.004 ^c^	0.056 ± 0.001 ^b^	0.063 ± 0.001 ^a^	0.224 ± 0.017 ^c^	0.493 ± 0.043 ^c^	***
Day 14	0.033 ± 0.003 ^c^	0.044 ± 0.003 ^b^	0.059 ± 0.003 ^a^	0.272 ± 0.022 ^c^	0.505 ± 0.048 ^c^	***
Day 16	0.034 ± 0.003 ^c^	0.045 ± 0.002 ^b^	0.057 ± 0.001 ^a^	0.270 ± 0.017 ^c^	0.473 ± 0.033 ^c^	***
**Maltose (g/L)**						
Day 0	33.167 ± 0.123 ^b^	34.478 ± 0.109 ^a^	34.673 ± 0.153 ^a^	33.27 ± 0.123 ^b^	33.220 ± 0.123 ^b^	***
Day 2	7.212 ± 0.169 ^a^	6.402 ± 0.214 ^b^	6.231 ± 0.226 ^b^	7.120 ± 0.186 ^a^	7.220 ± 0.269 ^a^	**
Day 5	0.615 ± 0.047 ^a^	0.672 ± 0.042 ^a^	0.599 ± 0.002 ^a^	0.615 ± 0.073 ^a^	0.615 ± 0.080 ^a^	N.S.
Day 7	0.516 ± 0.042 ^b^	0.732 ± 0.027 ^a^	0.659 ± 0.035 ^a^	0.518 ± 0.042 ^b^	0.516 ± 0.022 ^b^	***
Day 9	0.526 ± 0.018 ^ab^	0.435 ± 0.024 ^b^	0.534 ± 0.047 ^a^	0.526 ± 0.038 ^ab^	0.536 ± 0.045 ^a^	**
Day 12	0.349 ± 0.043 ^b^	0.328 ± 0.045 ^b^	0.510 ± 0.045 ^a^	0.456 ± 0.015 ^a^	0.415 ± 0.031 ^ab^	**
Day 14	0.344 ± 0.016 ^c^	0.325 ± 0.009 ^c^	0.508 ± 0.020 ^a^	0.438 ± 0.040 ^b^	0.418 ± 0.023 ^b^	***
Day 16	0.215 ± 0.036 ^b^	0.224 ± 0.030 ^b^	0.410 ± 0.035 ^a^	0.401 ± 0.033 ^a^	0.415 ± 0.031 ^a^	***
**Acetic acid (g/L)**						
Day 0	0 ^c^	0.006 ± 0.001 ^b^	0.008 ± 0.001 ^a^	0 ^c^	0 ^c^	***
Day 2	0 ^c^	0.028 ± 0.005 ^b^	0.101 ± 0.001 ^a^	0 ^c^	0 ^c^	***
Day 5	0 ^c^	0.005 ± 0.003 ^b^	0.053 ± 0.002 ^a^	0 ^c^	0 ^c^	***
Day 7	0 ^c^	0.021 ± 0.005 ^b^	0.035 ± 0.003 ^a^	0 ^c^	0 ^c^	***
Day 9	0.010 ± 0.005 ^b^	0.017 ± 0.004 ^ab^	0.028 ± 0.001 ^a^	0.011 ± 0.005 ^b^	0.012 ± 0.005 ^b^	**
Day 12	0.013 ± 0.003 ^c^	0.035 ± 0.002 ^b^	0.044 ± 0.003 ^ab^	0.021 ± 0.005 ^c^	0.049 ± 0.005 ^a^	***
Day 14	0.022 ± 0.005 ^c^	0.033 ± 0.002 ^b^	0.063 ± 0.002 ^a^	0.023 ± 0.004 ^c^	0.030 ± 0.002 ^bc^	***
Day 16	0.025 ± 0.005 ^c^	0.036 ± 0.002 ^b^	0.063 ± 0.002 ^a^	0.023 ± 0.004 ^c^	0.026 ± 0.001 ^c^	***
**Glycerol (g/L)**						
Day 0	0 ^b^	0.040 ± 0.002 ^a^	0.041 ± 0.003 ^a^	0 ^b^	0 ^b^	***
Day 2	0 ^b^	0.870 ± 0.033 ^a^	0.886 ± 0.013 ^a^	0 ^b^	0 ^b^	***
Day 5	0.863 ± 0.044 ^a^	0.857 ± 0.027 ^a^	0.895 ± 0.026 ^a^	0.865 ± 0.044 ^a^	0.866 ± 0.044 ^a^	N.S.
Day 7	0.856 ± 0.015 ^a^	0.850 ± 0.009 ^a^	0.865 ± 0.041 ^a^	0.855 ± 0.015 ^a^	0.858 ± 0.015 ^a^	N.S.
Day 9	0.849 ± 0.024 ^a^	0.862 ± 0.036 ^a^	0.855 ± 0.005 ^a^	0.852 ± 0.024 ^a^	0.850 ± 0.024 ^a^	N.S.
Day 12	0.858 ± 0.04 ^a^	0.862 ± 0.027 ^a^	0.860 ± 0.046 ^a^	0.854 ± 0.028 ^a^	0.884 ± 0.039 ^a^	N.S.
Day 14	0.857 ± 0.022 ^a^	0.859 ± 0.050 ^a^	0.865 ± 0.040 ^a^	0.867 ± 0.041 ^a^	0.862 ± 0.011 ^a^	N.S.
Day 16	0.856 ± 0.033 ^a^	0.853 ± 0.005 ^a^	0.867 ± 0.017 ^a^	0.850 ± 0.032 ^a^	0.873 ± 0.015 ^a^	N.S.

Values are expressed as the average of three measurements. Abbreviations: S.S., statistical significance. wort samples: CTR, wort without mushroom powder; PRE5, wort with 5 g/L of mushroom powder added before fermentation; PRE10, wort with 10 g/L of mushroom powder added before fermentation; POST5, wort with 5 g/L of mushroom powder added after fermentation; POST10, wort with 10 g/L of mushroom powder added after fermentation. Data within a line followed by the same letter are not significantly different according to Tukey’s test. Symbols: ***, *p* < 0.001; **, *p* < 0.01; *, *p* < 0.05; N.S., not significant.

**Table 2:** Physicochemical properties of beers.

Trial	Wort Extract	Real Extract	Apparent Extract	Alcohol	Real Attenuation	Energy	Specific Gravity	Density	pH	Color
	(*w*/*w*)	(*w*/*w*)	(*w*/*w*)	(%)	(%)	(kcal)		(g/mL)		EBC C.U.
CTR	11.42 ± 0.27 ^b^	5.15 ± 0.03 ^b^	3.69 ± 0.04 ^b^	4.14 ± 0.01 ^d^	56.5 ± 0.01 ^c^	42 ± 0.9 ^d^	1.015 ± 0.002 ^a^	1.013 ± 0.003 ^a^	4.13 ± 0.04 ^c^	3.82 ± 0.12 ^d^
PRE5	11.59 ± 0.22 ^b^	5.21 ± 0.03 ^b^	3.72 ± 0.07 ^b^	4.22 ± 0.04 ^cd^	56.6 ± 0.02 ^c^	43 ± 0.4 ^cd^	1.015 ± 0.005 ^a^	1.013 ± 0.003 ^a^	4.26 ± 0.02 ^b^	5.40 ± 0.63 ^cd^
PRE10	11.81 ± 0.28 ^ab^	5.34 ± 0.04 ^a^	3.84 ± 0.03 ^a^	4.28 ± 0.01 ^c^	56.3 ± 0.03 ^c^	44 ± 0.5 ^bc^	1.015 ± 0.003 ^a^	1.013 ± 0.003 ^a^	4.40 ± 0.04 ^a^	6.56 ± 0.45 ^b^
POST5	12.09 ± 0.34 ^ab^	4.67 ± 0.04 ^c^	2.96 ± 0.02 ^c^	4.90 ± 0.01 ^b^	62.9 ± 0.02 ^b^	45 ± 0.6 ^ab^	1.012 ± 0.001 ^a^	1.010 ± 0.001 ^a^	4.37 ± 0.03 ^a^	5.63 ± 0.34 ^bc^
POST10	12.47 ± 0.38 ^a^	4.33 ± 0.04 ^d^	2.46 ± 0.02 ^d^	5.37 ± 0.01 ^a^	66.8 ± 0.03 ^a^	46 ± 0.7 ^a^	1.010 ± 0.001 ^a^	1.008 ± 0.002 ^a^	4.44 ± 0.04 ^a^	11.85 ± 0.55 ^a^

Values are expressed as the average of three measurements. Abbreviations: *w*/*w*, wight/weight; EBC C.U., European Brewing Convention Color Unit. Beer samples: CTR, beer without mushroom powder; PRE5, beer with 5 g/L of mushroom powder added before fermentation; PRE10, beer with 10 g/L of mushroom powder added before fermentation; POST5, beer with 5 g/L of mushroom powder added after fermentation; POST10, beer with 10 g/L of mushroom powder added after fermentation. Data within a column followed by the same letter are not significantly different according to Tukey’s test. Mean values with different letters (a, b, c, d) within the same column are statistically different (*p*-value < 0.05).

## Data Availability

Data sharing not applicable.
